# Good clinical outcome for the majority of younger patients with hip fractures: a Swedish nationwide study on 905 patients younger than 50 years of age

**DOI:** 10.1080/17453674.2021.1876996

**Published:** 2021-01-22

**Authors:** Oscar Thoors, Carl Mellner, Margareta Hedström

**Affiliations:** a Department of Clinical Science, Intervention and Technology (CLINTEC), Karolinska Institutet, Stockholm;; b Department of Orthopedics, Eskilstuna Hospital;; c Department of Orthopedics, Karolinska Hospital Stockholm, Sweden

## Abstract

Background and purpose — Studies regarding hip fractures in young patients are rare since the patient population is small. We assessed clinical outcomes 4 months after hip fracture in patients < 50 years of age and whether there were differences between sexes and different age groups.

Patients and methods — We included adult patients < 50 years with a hip fracture between January 1, 2014 and December 31, 2018. Baseline data were extracted from the Swedish Registry for Hip Fracture Patients and Treatment (RIKSHÖFT) and mortality data was obtained from Statistics Sweden. The outcome variables were change of walking ability, pain in fractured hip, use of analgesics, living conditions, and mortality rate at 4 months.

Results — Of the 905 patients included, 72% were men and femoral neck fractures were most common (58%). 4 months after surgery, 23% used a walking aid and 7% reported severe pain. Women reported slightly more pain and higher usage of analgesics. Patients aged 40–49 reported higher usage of analgesics than patients aged 15–39, although the latter group reported more pain. Nearly all of those who lived independently before fracture did so at 4 months. The mortality rate was < 1%.

Interpretation — Most patients did not use any walking aid and few had severe pain at 4 months. Furthermore, a hip fracture is not a life-threatening event in a patient < 50 years. The living conditions did not change for those who lived independently before the fracture.

Numerous patients suffer from disability after a hip fracture and the 4-month mortality rate has recently been reported to be as high as 16% in patients older than 65 years of age (Greve et al. 2020). However, patients with hip fractures do not form a uniform entity. Of all patients with hip fractures, 2–11% are below 50 years of age (Rogmark et al. 2018). Consequently, most studies merely consider clinical outcome and mortality in the elderly and the generalizability to patients less than 50 years is therefore limited. The few previous studies have shown that the outcome of non-elderly patients is not as gruesome as in the elderly, but some report that the outcome in young patients is rather poor; however, in those studies the age limit for the non-elderly was set at less than 60 or 65 years of age and not 50 (Dargan et al. 2016, Ekegren et al. 2016, Rogmark et al. 2018). In this study we describe the < 50 years of age group and assess clinical outcomes 4 months after surgery and compare clinical outcome between sexes and between different age-groups.

## Patient and methods

### Study design

This nationwide cohort was based on data from all patients between 15 and 49 years of age, who had been operated on for a hip fracture between January 1, 2014 and December 31, 2018. All data was prospectively registered in the Swedish Registry for Hip Fracture Patients and Treatment (RIKS­HÖFT).

### Source of data and terminology

RIKSHÖFT has registered hip fractures patients (> 15 years of age) in Sweden since 1988 and has an estimated coverage of 80–90% for the years studied (Meyer et al. 2020). The date of death was obtained through record linkage with the National Death Register, Statistics Sweden. Baseline data on all patients included age, sex, waiting time until surgery (hours between arrival at hospital and start of surgery), cognitive function, divided into 3 categories: no cognitive dysfunction; signs of confusion; a diagnosis of dementia. Based on RIKSHÖFT, fracture types were grouped into non-displaced, displaced femoral neck fractures, and basicervical fractures merged to femoral neck fractures (FNF) and into trochanteric and subtrochanteric fractures. Surgical methods were registered as 2 or more screws, sliding hip screw, intramedullary nail, total hip arthroplasty, hemiarthroplasty, or nonoperative treatment. Use of a walking aid was categorized as: no use of walking aid, 1 crutch, 2 crutches, walker, wheelchair. Coming from was categorized as: own home, group/service housing, full-service unit, rehabilitation clinic, emergency hospital, or other. Comorbidity was measured through ASA classification, which was assessed preoperatively by the local anesthesiologist or the local orthopedist on call as part of standard preoperative practice.

All patients either received a questionnaire from the register or were called by phone 4 months after the operation. The following variables were included: use of walking aid or not, living independently or not, pain in fractured hip, and use of analgesics because of the hip fracture. Living independently was defined as patients residing in their own homes, with or without assistance from home care aids. In RIKSHÖFT, pain is assessed in 6 different categories, but was in this study merged into 3: no/transient pain, mild/intermittent pain, and severe/continuous pain. The kind of analgesics used were not specified—simply a yes or no answer.

Patients were divided into subgroups: those aged 15–39 and 40–49, and sexes. Patients who used a walking aid before the fracture were excluded in the analysis of use of walking aid 4 months after surgery. Only patients living at home before the fracture were analyzed regarding their living conditions 4 months after surgery (Figure).

**Figure:**
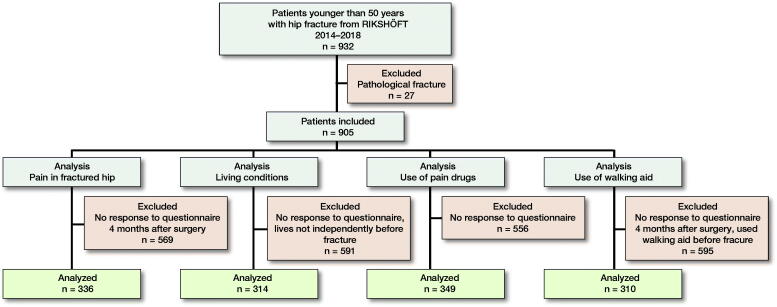
Patients included in the study.

### Statistics

Descriptive data was presented with means (SD), percentages, and range. Non-normally distributed independent data were tested for differences with a Mann–Whitney–Wilcoxon test. Contingency tables were used for categorical data and tested for differences using the chi-square test. A p-value < 0.05 was considered statistically significant. Statistical calculations were performed using IBM® SPSS Statistics® for Windows version 25.0 (IBM Corp, Armonk, NY, USA).

### Ethics, data sharing, funding, and potential conflict of interests

The study was conducted in accordance with the ethical principles of the Helsinki Declaration and approved by the Regional Ethics Committee of Stockholm (DNr: 2017/1088-31). This study was based on sensitive individual-level data protected by the Swedish personal data act. Data can therefore only be shared after ethical approval and the consent of the principal investigator. The study was supported by grants provided by Region Stockholm (ALF project). The authors declare no conflicts of interest.

## Results

905 patients were included in the study (Figure) and represented 1.2% of all hip fractures registered during these years in RIKSHÖFT. The median age was 42 years (15–49) (Table 1) and 72% were men.

**Table 1.** Baseline data for all patients younger than 50 years with a hip fracture. Values are count (%) unless otherwise specified

Factor	All patients	Men	Women	p-value **^a^**
Overall sample	905	652 (72)	253 (28)	
Age, median	42	42	43	
range	15–49	15–49	18–49	
Age groups				0.5
15–39	360 (40)	264 (40)	96 (38)	
40–49	545 (60)	388 (60)	157 (62)	
Fracture-type				0.07
Cervical (FNF)	528 (58)	365 (56)	163 (64)	
Trochanteric	234 (26)	179 (27)	55 (22)	
Subtrochanteric	143 (16)	108 (17)	35 (14)	
ASA score **^b^**				< 0.001
1	446 (50)	347 (54)	99 (40)	
2	293 (33)	199 (31)	94 (38)	
3	135 (15)	91 (14)	44 (18)	
4	16 (2)	6 (1)	10 (4)	
Mental status **^c^**				0.4
No cognitive				
dysfunction	631 (95)	461 (96)	170 (93)	
Signs of confusion	29 (4.5)	18 (4.7)	11 (6)	
Diagnosed with				
dementia	4 (0.5)	2 (0.3)	2 (1)	
Walking-aid				0.04
No use of walking aid	795 (88)	585 (89)	210 (82)	
1 crutch	13 (1.5)	22 (3.5)	13 (5)	
2 crutches	14 (2)	10 (1.5)	9 (3.5)	
Walking with walker	27 (3)	19 (3)	14 (6)	
Wheelchair	48 (5.5)	19 (3)	9 (3.5)	
Coming from				0.3
Own home	801 (89)	582 (89)	219 (87)	
Group/service housing	34 (3.8)	19 (3)	15 (6)	
Full-service unit	16 (2)	12 (2)	4 (1)	
Rehabilitation clinic	1 (0.1)	1 (0.2)	0	
Emergency hospital	41 (4)	28 (4)	13 (5)	
Other	12 (1.1)	10 (1)	2 (1)	

**^a^**p-values were calculated using Mann–Whitney U-test for variables on continuous or ordinal scale. Chi-square test for categorical variables.

**^b^**Missing = 15.

**^c^**Missing = 241.

### Patient and descriptive data

The majority (89%) of all patients lived independently before the fracture (Table 1). Women had a lower proportion of ASA-1 compared with men. 12% used a walking aid before the fracture, women more than men (18% and 11%, respectively). The most common type of fracture overall was an FNF (58%) (Table 1). Subtrochanteric fractures were more common in the younger age group compared with the older age group (21% vs. 12%) and the FNF were less common in the younger age group compared with the older age group (55 vs. 61%) (Table 2). 45% were treated with 2 screws or more, 25% were treated with intramedullary nail, 26% were treated with sliding hip screw, 3% were treated with total or hemi­arthroplasty, and 1% received nonoperative treatment.

**Table 2.** Baseline data on all patients with a hip fracture aged 15–39 and 40–49 years of age. Values are count (%)

Factor	15–39	40–49	p-value **^a^**
Sex			0.5
Men	264 (73)	388 (71)	
Women	96 (27)	157 (29)	
Fracture type			0.002
Cervical (FNF)	198 (55)	330 (61)	
Trochanteric	86 (24)	148 (27)	
Subtrochanteric	76 (21)	67 (12)	
ASA score			< 0.001
1	206 (59)	240 (45)	
2	102 (29)	191 (35)	
3	39 (11)	96 (18)	
4	3 (1)	13 (2)	
Mental status			0.8
No cognitive dysfunction	231 (96)	400 (95)	
Signs of confusion	10 (4)	19 (4)	
Diagnosed with dementia	0	4 (1)	
Walking aid			0.003
No use of walking aid	332 (93)	463 (86)	
1 crutch	3 (0.8)	10 (1.8)	
2 crutches	2 (0.5)	12 (2.2)	
Walking with walker	3 (0.8)	24 (4.5)	
Wheelchair	17 (4.9)	31 (5.5)	
Coming from			0.4
Own home	323 (90)	478 (88)	
Group/service housing	9 (2.5)	25 (4.5)	
Full-service unit	4 (1)	12 (2)	
Rehabilitation clinic	0 (	1 (0.2)	
Emergency hospital	19 (5)	22 (4)	
Other	5 (1.5)	7 (1.3)	

**^a^**p-values were calculated using Chi-square test

### Clinical outcomes at 4-month follow-up

310 patients were analyzed regarding the use of a walking aid and 336 patients regarding pain in the hip at 4-month follow-up (Figure, Table 3). Of all patients walking without walking aids before fracture (88%), 77% did not use any walking aid 4 months after surgery. Severe pain was present in 8% of the women compared with 6% in men. Women also used more analgesics because of hip pain (28%) compared with men (15%) (Table 3). Of those who lived independently in their own home before the fracture (89%), 98% still lived independently at 4 months. There was no statistically significant difference between the 2 age groups regarding use of a walking aid. In those aged 15–39, some pain was present in a higher degree compared with patients aged 40–49 (78% and 66%, respectively). Patients aged 40–49 used more analgesics than their younger counterparts (15% versus 9%) (Table 4).

**Table 3.** Clinical outcomes 4 months after surgery in hip fracture patients younger than 50 years of age, living independently, and walking without a walking device. Values are count (%)

Factor	All	Men	Women	p-value **^a^**
Walking-aid **^b^**				0.2
No use of walking aid	240 (77)	167 (80)	73 (73)	
Use of walking aid	70 (23)	43 (20)	27 (27)	
Pain				0.2
No/transient pain	101 (30)	75 (33)	26 (23)	
Mild/intermittent pain	212 (63)	136 (61)	76 (69)	
Severe/substantial pain	23 (7)	14 (6)	9 (8)	
Analgesics because of fracture				0.007
Yes	68 (20)	36 (15)	32 (28)	
No	281 (80)	197 (85)	84 (72)	
Living conditions **^c^**				0.1
Lives independently	309 (98)	206 (98)	103 (100)	
Lives not independently	5 (2)	5 (2)	0 (0)	
Deaths	6	2	4	

**^a^**p-values were calculated using Chi-square test.

**^b^**Patients who walked without walking aid before the fracture.

**^c^**Patients who lived independently before the fracture.

**Table 4.** Differences between age groups in clinical outcomes 4 months after surgery, use of walking aid and living conditions. Values are count (%)

Factor	All	15–39	40–49	p-value **^a^**
Walking aid **^b^**				0.09
No use of walking aid	240 (77)	95 (83)	145 (74)	
Use of walking aid	70 (23)	20 (17)	50 (26)	
Pain				0.07
No/transient pain	101 (30)	25 (22)	76 (34)	
Mild/intermittent pain	212 (63)	80 (70)	132 (60)	
Severe/substantial pain	23 (7)	9 (8)	14 (6)	
Analgesics because of fracture				0.04
Yes	68 (20)	18 (9)	50 (15)	
No	281 (80)	179 (91)	281 (85)	
Living conditions **^c^**				0.7
Lives independently	309 (99)	105 (99)	204 (99)	
Lives not independently	5 (2)	1 (1)	4 (1)	
Deaths	6	2	4	

**^a-c^**See Table 3

### Mortality at 4 months

The mortality rate was 0.7%: 2 men (aged 44, 45), and 4 women (aged 31, 39, 2 aged 49) (Tables 3 and 4).

### Non-responders

A non-response analysis of the differences in baseline data between patients with outcome data and those without showed a higher proportion of men and those aged 15–39 in the latter group (Table 5, see Supplementary data).

## Discussion

This register-based study identified that 1.2% of all patients with a hip fracture were younger than 50 years of age. The majority did not use any walking devices at 4 months postoperatively, 7% reported severe hip pain, and 20% used analgesics. The living conditions did not change considerably for patients who lived independently before the fracture and less than 1% were deceased 4 months after surgery.

A vast majority of this young age group with hip fracture were men, in accordance with other studies (Al-Ani et al. 2013, Lin et al. 2014, Mattisson et al. 2018), and with fractures in general (Farr et al. 2017). FNF was the most common fracture type, nearly 60%, in contrast to the known even distribution of fracture types in the elderly (RIKSHÖFT annual report 2019). Studies on fracture types in younger patients are scarce, but 2 earlier studies from Scotland and Taiwan also reported FNF being most common among the youngest (Robinson et al. 1995, Wang et al. 2017).

23% used some walking aids 4 months postoperatively as compared with a previous study on patients aged 65 or younger where the numbers were similar but after 12 months (Dargan et al. 2016). 7% reported severe pain in their fractured hip at 4-month follow-up, slightly more women, and women also used more analgesics. The reason for this difference is unclear; it may be due to sex differences in pain expression (Skogö Nyvang et al. 2019) or coping strategies between sexes (Racine et al. 2012). A higher proportion of the youngest patients aged 15–39 reported more pain in their fractured hip than elderly patients. We found only 1 study that assessed hip pain as a clinical outcome after hip fracture in patients aged 21–56 (Jain et al. 2004). But comparisons with this study are difficult, since they reported that only 6 out of 23 patients had pain 6 months after their hip fracture. The assessment of pain is often included in different evaluation scores, such as the Harris Hip Score, Arnold Evaluation Score and Merle D’aubergine scoring system, without details regarding pain level, thus also making comparisons difficult with those studies (Sprague et al. 2015, Rogmark et al. 2018).

Whether trauma mechanism influenced and explained postoperative pain 4 months after the fracture is unclear; we do not have any data regarding trauma mechanism in this study. However, 1 recent study showed that the majority of patients aged 20–49 with a hip fracture actually suffered from a low-energy trauma (fall from standing height or less) (Al-Ani et al. 2013), similar to a Swedish study on younger men with distal radius fractures (Egund et al. 2016). The trauma mechanism behind a hip fracture in the youngest might have changed since 1982, when Zetterberg et al. (1982) reported that high-energy trauma was the leading trauma mechanism

In our study, less than 1% of 905 patients were deceased at 4 months, to be compared with 16% in patients > 65 years with a hip fracture (Söderqvist et al. 2009, Greve et al. 2020). Other studies have shown a similar low mortality rate after a hip fracture in young patients (Robinson et al. 1995, Lin et al. 2014).

Living conditions are considered to be a good measure in overall recovery after a hip fracture and have a positive impact on the quality of life (Boelhouwer 2002). In this study, we found that 98% of those living independently before fracture had return to their own living arrangements 4 months after surgery, contrary to the situation for many elderly patients (RIKSHÖFT annual report 2019).

### Strength and limitations

One strength is the prospective nationwide design based on RIKSHÖFT, including a larger number of patients. Another is the many variables included in the baseline data, which provided a broad picture of the young patient with a hip fracture. The weakness of this study is the low response rate to the questionnaire regarding clinical outcomes. However, other Swedish national registries have a similar low response rate to questionnaires (Swedish National Knee Ligament Registry, 2018, Swedish Fracture Register, 2019). The low response rate may have introduced a selection bias, thus reducing the external validity of this study, although a non-response analysis showed that the only differences were that men and patients aged 15–39 had a lower response rate (Table 5, see Supplementary data).

In conclusion, most young patients with a hip fracture had good walking function at 4 months and few reported severe pain. The mortality rate was low and living conditions for those living independently before the fracture did not change substantially.

## Supplementary Material

Supplemental Material

## Supplementary data

Table 5 is available as supplementary data in the online version of this article, http://dx.doi.org/10.1080/17453674.2021.1876996
