# Supplementary material for: Good clinical outcome for the majority of younger patients with hip fractures: a Swedish nationwide study on 905 patients younger than 50 years of age
Source: Acta Orthop. 2021 Jan 22;92(3):292–6. doi: 10.1080/17453674.2021.1876996 (PMC8231413; doi:10.1080/17453674.2021.1876996)
Supplement: Supplemental Material [file IORT_A_1876996_SM0410.pdf]

Supplementary data

Table 5. Non-response analysis: difference between patients who did and did not respond to the questionnaire 4 months after surgery. Values are count (%) unless otherwise specified

Factor	Without outcome data	With outcome data	p-value
Sex			< 0.001
Men	413 (66)	210 (32)	
Women	131 (57)	100 (43)	
Age, median (range)	41 (15–49)	43 (15–49)	
Age groups			0.007
15–39	236 (65)	124 (35)	
40–49	308 (57)	237 (43)	
ASA score			0.09
1	276 (52)	170 (47)	
2	175 (33)	118 (33)	
3	72 (14)	63 (17)	
4	6 (1)	10 (3)	
Coming from			0.09
Living independently	488 (90)	313 (87)	
Group home	13 (2.5)	21 (6)	
Full-service group home	9 (2)	7 (2	
Rehabilitation clinic	0	1 (0.5)	
Emergency hospital	25 (4.5)	16 (4.5)	
Other	9 (1)	3 (1)	
Mental status			0.4
No cognitive dysfunction	363 (96)	268 (94)	
Signs of confusion	15 (3.9)	14 (5)	
Diagnosed with dementia	1 (0.1)	3 (1)	
Fracture type, n (%)			0.4
Cervical	311 (57)	217 (60)	
Trochanteric	140 (26)	94 (26)	
Subtrochanteric	93 (17)	50 (14)	
Housing, days median (range)	4 (1–38)	4 (0–77)	
